# Effects of a cognitive-behavioral exposure-based body image therapy for overweight females with binge eating disorder: a pilot study

**DOI:** 10.1186/s40337-017-0174-y

**Published:** 2017-12-21

**Authors:** Merle Lewer, Joachim Kosfelder, Johannes Michalak, Dorothea Schroeder, Nadia Nasrawi, Silja Vocks

**Affiliations:** 1 Department of Clinical Psychology and Psychotherapy, Ruhr-Universität Bochum, Bochum, Germany; 2 Department of Social and Cultural Sciences, University of Applied Sciences, Duesseldorf, Germany; 3 Department of Psychology and Psychotherapy, Witten/Herdecke University, Witten, Germany; 4 Department of Clinical Psychology and Psychotherapy, Osnabrueck University, Knollstrasse 15, D-49069 Onabrück, Germany

**Keywords:** Binge eating disorder, Body image disorder, Cognitive-behavioral therapy, Digital photo distortion technique, Exposure therapy

## Abstract

**Background:**

Although not part of the diagnostic criteria of the DSM-5, body image disturbance seems to be a relevant feature of Binge Eating Disorder (BED) as well as of other eating disorders such as Anorexia Nervosa (AN) or Bulimia Nervosa (BN). Hence, the aim of the present pilot study was to assess the changeability of body image disturbance in a sample of overweight females with BED by a cognitive-behavioral treatment, directly addressing body image disturbance.

**Method:**

Overweight females (*N* = 34) with BED were randomized to a manualized body image therapy or a waiting-list control group. The final sample consisted of *n* = 15 participants in the intervention group and *n* = 19 in the control group due to two drop-outs in the control condition. Before and after the intervention or the waiting period, respectively, participants filled out a questionnaire battery assessing several body image and eating disorder related features. To quantify the perceptual component of body image disturbance, a digital photo distortion technique based on a picture of each participant taken in a standardized suit was applied.

**Results:**

In a two-way ANOVA, significant Time × Group interactions were found for eating and shape concerns, drive for thinness, body dissatisfaction, depressiveness and low self-esteem. Follow-up *t*-tests indicated a significant symptom reduction of a generally high magnitude in the intervention group. No significant changes concerning body checking and the estimations of one’s own “real”, “felt” and “ideal” body dimensions were found.

**Conclusion:**

The strong symptom reduction in the cognitive-affective component of body image disturbance indicates that an exposure-based cognitive-behavioral body image intervention is a promising treatment module for overweight females with BED, but future research with a larger sample size is needed to quantify possible changes in all components of body image.

## Lay summary

A disturbed body image is an important criterion of eating disorders, such as anorexia and bulimia nervosa. There are increasing hints that people who suffer from binge eating disorder might have a disturbed body image as well. In this study, we tested the changeability of a disturbed body image in binge eating disorder. It was shown that the cognitive-emotional component, that is, the way one thinks and feels about one’s body, as well as factors such as depression and self-esteem improved significantly during a group program which was designed to change body image disturbance in persons with binge eating disorder.

## Background

Binge eating disorder (BED) is characterized by recurring episodes of binge eating during which a large amount of food is consumed in a certain time frame with a co-occurring feeling of loss of control over the amount or type of food consumed. These binge-eating episodes are accompanied for example by the individual feeling ashamed while eating, eating fast and being disgusted by themselves. In the fifth edition of the *Diagnostic and Statistical Manual of Mental Disorders* (DSM-5) [1], BED is recognized as an autonomous diagnosis in the eating disorders section. It is estimated that about 1–4% of women in Europe suffer from BED [2]. In contrast to bulimia nervosa (BN) and binge-eating/purging type of anorexia nervosa (AN), binge eating episodes are not accompanied by regular inappropriate compensatory behaviors for weight regulation as, for example, self-induced vomiting or laxative abuse. About 70% of patients with BED are at least overweight or suffer from comorbid obesity [3, 4], which might lead to serious medical obesity-related complications such as the metabolic syndrome including dyslipidemia, hypertension and type-2 diabetes [5, 6]. Especially, morbid obesity (body mass index >35 kg/m^2^) is associated with greater health risks [7]. Beyond that, BED also results in a reduction in psychosocial functioning and quality of life [8, 9] and leads to an elevated risk of comorbid psychiatric disorders such as major depression, anxiety disorders and suicide risk, regardless of co-occurring obesity [10].

As opposed to AN and BN, the DSM-5 criteria for BED do not contain a body-image related criterion. However, there is growing evidence for the existence of body image disturbance in BED as well [11–13], at least for the application of it as a specifier or severity criterion [14]. Nevertheless, a number of studies indicate that body image disturbance, which is often described according to three components of body image: the cognitive-affective, the behavioral and the perceptual [15], is not only characteristic of AN and BN, but also seems to be present in BED, even when considering the often co-occurring overweight. Stronger weight and shape concerns were found in obese patients with BED compared to obese participants without BED [16–20], indicating a disturbance in the cognitive-affective component of body image, which seems not to be solely due to being overweight. Furthermore, it was found that overvaluation of shape and weight was related to a more pronounced eating disorder pathology, but was not associated with the body mass index (BMI) [21]. Within weight and shape concerns, the cognitive-affective component of body image disturbance is characterized by a negative evaluation of one’s own body [22, 23]. Studies addressing this aspect in BED have yielded inconsistent results. Whereas some studies found that obese patients with BED displayed a higher degree of body dissatisfaction than obese participants without BED [18, 24], other studies did not find group differences [13, 25–27]. A laboratory study indicated that patients with BED display lower mood levels than healthy controls when they looked at their own body [28], hinting to elevated levels of body dissatisfaction. Furthermore, the cognitive-affective component of body image disturbance covers the internalization and awareness of the slenderness ideal of Western societies which is assumed to be a correlate and predictor of disordered eating behavior [29–32].

The behavioral aspect of body image disturbance comprises specific body-related behaviors such as body image avoidance (e.g., not looking in the mirror) [33] or body checking (e.g., measuring the circumference of various body parts) [34]. To date, this aspect of BED has been investigated only in three studies. Reas, Grilo, Masheb and Wilson [35] found that in overweight females and males with BED, body-related avoidance and checking behavior was positively correlated with the extent of weight and shape concerns. However, as the authors did not compare their participants with a control group one could not distinguish whether the higher degree of body checking and body avoidance was related to the binge eating pathology itself or to being overweight. In the other studies, Legenbauer et al. [36] and Lewer et al. [13] did not find any significant differences between obese patients with and without BED concerning body-related avoidance or checking behavior.

Finally, the perceptual component of body image disturbance encompasses the misjudgment of one’s own body size [37]. Many studies with patients with AN and BN have been conducted assessing body size misperception and indicated that both patient groups overestimate their own body dimensions, while healthy females tend to slightly underestimate them [38, 39]. In one study, it was found that obese patients with BED felt significantly (9%) larger than they really were [36]. However, no significant differences were found between this group and obese patients without BED. Similarly, Sorbara and Geliebter [24] did not find differences between obese patients with and without BED in terms of the estimation of one’s own body size. In a recent study, Lewer et al. [13] showed that obese individuals with BED wished for a slimmer ideal figure than obese participants without an eating disorder, but did not find any differences concerning the perceived real and felt figure.

Previous research indicates that body image disturbance plays a central role in the etiology, maintenance and relapse process of eating disorders [40–45]. Moreover, the extent of body image disturbance is correlated with low self-esteem and depression [46, 47]. These findings raise the question of how body image disturbance in eating disorders can be treated effectively. For AN and BN, several studies demonstrated the effectiveness of cognitive-behavioral interventions to improve body image disturbance [48–56].

In contrast, investigating the convertibility of a disturbed body image in overweight and obese patients with BED has been neglected, although this aspect is of high relevance for this patient group, as previous research has demonstrated the high risk of failure of weight reduction treatments in BED [57], and that body dissatisfaction is a risk factor for the onset of binge eating [58]. Furthermore, Sonneville et al. (2012) [59] could show that being satisfied with one’s body prevents weight gain and binge eating in adolescent girls, whereas girls who suffered from body dissatisfaction had the tendency to gain more weight over time. In the only study on body image therapy for overweight females with BED to date [60], two cognitive-behavioral treatment components, body exposure and cognitive restructuring, were compared. The study revealed positive results, as both treatment components were shown to be effective in terms of reduction of dysfunctional assumptions about shape and weight as well as body dissatisfaction. However, no significant group differences were found between the two conditions. As no untreated control group was included in the trial, interpretation of the findings is difficult, especially when considering the generally high degree of spontaneous fluctuations of BED symptomatology [61]. In addition, the perceptual aspect of body image disturbance including the mental representation of one’s body size as well as the behavioral component including direct measures of body avoidance and body checking have not been studied in previous research regarding their convertibility.

Based on these considerations, the present pilot study used a randomized-controlled parallel-group design with a waiting-list group as control condition in order to test for the effects of a short manualized exposure-based cognitive-behavioral group intervention focusing on overcoming body image disturbance on the various components of body image disturbance in a sample of overweight females with BED. It was assumed that the intervention group would show significant changes in all components of body image disturbance as opposed to the waiting-list control group.

## Methods

### Participants

Inclusion criteria for participation in the present study were a diagnosis of BED according to the research criteria of the fourth *Diagnostic and Statistical Manual of Mental Disorders* [62], being overweight (BMI > 25 kg/m^2^), female sex and an age between 18 to 60 years. Persons were excluded if they suffered from a personality disorder, displayed suicidal tendencies, showed deliberate self-harm behavior or were pregnant. Diagnoses of personality disorders were excluded, because the treatment program included potentially stressful interventions such as exposure, which can provoke intense tension and eventually self-harm behavior. The goal of the study was to investigate a new intervention for BED in a preferably homogenous sample. Further exclusion criteria were a current psychotherapy and the intake of psychotropic drugs, to avoid confounding effects when analyzing the results of the treatment program. Participants were recruited from the waiting list of the Mental Health Research and Treatment Center of the Ruhr-Universität Bochum and by newspaper announcements. After a brief telephone screening, participants were invited to a diagnostic session. In this session, the diagnoses were assessed by experienced postgraduate clinical psychologists using the *Structured Clinical Interview for DSM-IV* (SCID) [63, 64] and the *Eating Disorder Examination*–*Interview* (EDE) [65, 66]. Personality disorders were assessed using the screening questionnaire of the SCID-2 [67] and if necessary the SCID-2 interview. In order to determine the BMI, patients’ heights were measured and the participants were weighed using a standardized balance. Thirty-six females fulfilled the inclusion criteria and agreed to participate. Participants were randomized either to the body image therapy condition (intervention group, IG; *n* = 15) or to the waiting list control condition (control group, CG; *n* = 21). From the control condition, *n* = 2 patients dropped out of the study prematurely, resulting in a final sample size of *n* = 15 for the IG and *n* = 19 for the CG. The study protocol was reviewed and approved by the local Ethics Committee of the Ruhr-Universität Bochum.

### Self-report questionnaires

To quantify the changes of various aspects of body image disturbance and related constructs in order to determine outcome, the following self-report questionnaires, which are widely used in eating disorders research, were administered to the patients at pre- and post-test: From the Eating Disorder Inventory-2 [68, 69], the subscales Drive for thinness, Bulimia and Body dissatisfaction were included. The subscale Drive for Thinness consists of six items, the scale Bulimia contains seven items and the scale Body Dissatisfaction is assessed with nine items. Each item is answered on a 6-point scale (1 = “never” to 6 = “always”). In line with other studies with patients with BED [13], internal consistencies in the present study (Cronbach’s alpha) were α = .70 for Drive for Thinness, α = .70 for Bulimia and α = .60 for Body Dissatisfaction, while in patients with AN and BN internal consistencies for these subscales range from α = .88 to .93 [69]. Furthermore, we used the Eating Disorder Examination–Questionnaire [66, 70] which consists of the four subscales Restraint, Eating concern, Weight concern and Shape concern. The subscales Restraint, Eating concern and Weight concern consist of five items each, and the subscale Shape concern is made up of eight items. The items are scored on a 7-point scale (0 = “attribute was not present” to 6 = “attribute was present every day”). The internal consistency of the scales varies from α = .51 for Restraint, Weight concern and Eating concern to α = .79 for Shape concern in our sample, other studies have displayed adequate internal consistency and retest-reliability of the scales, as well as good validity with the Eating Disorder Examination [70–72].

To assess body-related behavior, we used the *Body Checking Questionnaire* [73, 74]. Cronbach’s alpha in the present sample is α = .91, similar to other studies [74] and also showing good convergent validity [56]. The Body Checking Questionnaire consists of 23 items to assess how often people are involved in body checking behavior). The items are each scored on a scale from 1 (= “never”) to 5 (= “very often”).

Furthermore, the *Rosenberg Self-Esteem Scale* [75, 76] was included as a measure to assess the broader construct of self-esteem, which consists of ten items that are scored between 0 (= “never applies to me”) and 3 (= “always applies to me”). The internal consistency of the inventory is α = .81 for the present sample and ranges from α = .81 to α = .88 in other samples [75]. Finally, we used the *Beck Depression Inventory* [77] in order to assess the degree of depressiveness. The BDI comprises 21 items that are answered on a 4-point scale, with higher values indicating a greater level of depression. Cronbach’s α in the present study is α = .84, which is in line with other studies [13] and varies from α = .74 to α = .88 according to the authors [77]. Furthermore, the BDI has proven to be a reliable marker for stress [78].

### Digital photo distortion technique

In addition to the self-report questionnaires, the *Digital Photo Distortion Technique* [79] was applied in the present study in order to assess the perceptual component of body image disturbance, since optical distortion methods have been shown to have the highest construct and ecological validity of methods to assess body size misperception (e.g., for the situation of looking at oneself in a mirror) [37]. In comparison, analogue scales or image marking methods have the tendency to measure other skills (e.g., memory, size perception) than assessing the perceived body size. Furthermore, usually only single parts of the body or drawn figures are presented which is quite different from looking at oneself in the mirror or at one’s photograph [37]. In this study, a digital photo of each participant was taken from a frontal perspective while the participants were standing in front of a white wall wearing a standardized tight-fitting pink suit. The suit had short arms and legs and was available in several sizes. After that, the pictures were fed into the digital distortion system and were presented to the participants on a laptop computer screen. By pressing two arrow keys, patients could interactively adjust the width of the displayed picture of themselves, thus making their body appear thinner or larger. Pressing the keys once, the picture was distorted by +0.8% or −0.8%. Using this method, the participants were asked to indicate their “actual” (“What do you really look like?”), “felt” (“What do you feel you look like?”), and “ideal” (“What would you like to look like?”) body dimensions [80]. The questions appeared in a separate field under the distortable photograph. Patients were allowed to correct the degree of distortion of the photo as frequently as they liked. A value of 100% indicated the original size of the photo, values below 100% a distortion towards a slimmer, and values over 100% towards a larger body. Due to a possible anchor effect depending on the initial distortion of the presented stimulus [81, 82], each photograph was presented four times; twice initially distorted in the slimmer direction (80%) and twice in the larger direction (120%). Internal consistency for the three questions ranged from α = .90 to .94 in the present sample, which is in accordance with other studies [54].

### Body image therapy

The cognitive-behavioral body image therapy was conducted at the outpatient Mental Health and Research and Treatment Centre of the Ruhr-Universität Bochum. The intervention was carried out by two female clinical psychologists experienced in the treatment of eating disorders and trained in cognitive-behavioral therapy. The clinical psychologists received special training in conducting the group sessions by the author of the treatment manual [83], who also supervised the treatment process once in every three sessions. The special training thereby included intensive study and detailed discussion of the study manual. The body image therapy consisted of ten weekly sessions, each lasting for approximately 90 min. The intervention was manualized and the content of each of the sessions was standardized [54]. In the beginning of the intervention, the perceptive, cognitive-affective, and behavioral components of a disturbed body image were introduced to the patients. Based on this, an individual model of the development and maintenance of body image disturbance was derived for each participant, comprising of two sessions in total. In the following two sessions, dysfunctional body-related thoughts of each participant were identified and modified using several cognitive techniques such as the “Socratic dialogue” and positive self-instructions. In the following three sessions, body exposure was introduced by using mirrors and video feedback. In a first step, the focus was set on negatively evaluated and avoided body parts to achieve habituation and develop a realistic image of one’s own body. This was followed by tasks aiming at focusing on positive body areas to shift the attention away from a deficit-oriented body image. In order to reduce body avoidance and body checking behavior, additional exposure exercises were introduced and carried out in a further session in various real-life situations (e.g., visiting a public swimming pool). Afterwards, tasks aiming at enhancement of the frequency and hedonic qualities of potentially rewarding body-related activities were introduced in a following session and assigned as homework (e.g., dancing, visiting a sauna, using personal-care products such as body lotion). The final session dealt with relapse prevention. In order to further elaborate the contents of each of the sessions, homework was assigned regularly [84, 85].

### Procedure

After the structured diagnostic interview with the SCID and the EDE, and once the eligible patients with BED had been informed about the study and agreed to participate, they filled out the questionnaire battery. The digital photo distortion technique was applied on a separate day. An unrestricted randomization procedure was used. By casting a dice, participants were then randomized either to the IG or the CG. The intervention groups consisted of three to six participants. The waiting time for the CG was scheduled to be ten weeks, which equates to the length of the intervention period in the IG. After the intervention or the waiting time, respectively, participants of both groups answered the self-report questionnaire battery once again and the digital photo distortion technique was applied a second time.

### Statistical analysis

Data analysis was conducted using the SPSS 16 software. First, the two groups were compared in terms of age, BMI, the scores on the self-report questionnaires and the indices of the digital photo distortion technique at pre-test, using a two-sample *t*-test including Levene’s test of homogeneity of variances. To test for the treatment effects in a completer-analysis, a two-way ANOVA with the within-subjects factor Time (Pre versus Post) and the between-subjects factor Group (IG versus CG) was calculated. Dependent variables were the scores of the self-report questionnaires and the values assessed using the digital photo distortion technique. To control for confounding effects, comorbidity was inserted as a covariate. The significance level was fixed at *p* < .05 (two-tailed). Cohen’s *d* [86] was calculated to quantify the pre-post changes in the two groups. To follow up the Time × Group interactions of the ANOVA, repeated *t*-tests were calculated. To reduce the probability of α-error, a bonferroni correction was applied on the post-hoc t-tests.

## Results

### Comparison of the two groups at pre-test

At pre-test, the IG and CG did not differ in age (*t*(31) = −0.58; *p* = .561); however, the CG had a higher BMI than the IG (*t*(28) = −2.22; *p* = .034). As assessed by the SCID [63, 64], seven participants of the IG did not have any comorbid psychiatric disorders, six suffered from comorbid major depression, one reported panic disorder, and one participant displayed symptoms of specific phobia. Five participants of the CG reported no further diagnosis, *n* = 10 reported major depression, one participant suffered from comorbid panic disorder, two from social phobia and one from posttraumatic stress disorder. According to the questionnaire measures, the two groups did not differ in their scores on the subscales Drive for thinness (*t*(32) = 0.69; *p* = .494), Bulimia (*t*(32) = 1.33; *p* = .191) and Body dissatisfaction (*t*(32) = −4.70; *p* = .642) of the *Eating Disorder Inventory* -*2* and in the subscales Restraint (*t*(32) = 1.84; *p* = .075), Eating concern (*t*(32) = 0.67; *p* = .507), Weight concern (*t*(32) = −1.01; *p* = .920) and Shape concern (*t*(32) = 0.64; *p* = .949) of the *Eating Disorder Examination*–*Questionnaire* at pre-test. Furthermore, the groups did not differ in the general score of the *Body Checking Questionnaire* (*t*(32) = 1.87; *p* = .072). Similarly, no differences were found on the general scores of the *Rosenberg Self-Esteem Scale* (*t*(32) = 1.42; *p* = .165) and the *Beck Depression Inventory* (*t*(32) = −1.35; *p* = .201). In the *Photo Distortion Technique*, groups did not differ in terms of their estimates of their “real” (*t*(32) = −1.54; *p* = .133), “felt” (*t*(32) = −0.92; *p* = .361) and “ideal” body dimensions (t(32) = −0.31; *p* = .752) at pre-test.

### Comparison of the two groups in their changes from pre to post body image therapy

For time and group effects and time x group interactions see Table 1. For the BMI, the Time × Group-interaction was not significant.

**Table 1:** Means and standard deviations of pre- and post-scores of the questionnaire measures and the Photo Distortion Technique in the intervention group and in the control group

		Intervention group		Control group		Time			Group			Time × group		
Measure		M	SD	M	SD	F	df	p	F	df	p	F	df	p
Body Mass Index (kg/m^2^)														
	Pre	31.98	4.70	36.80	5.08	.098	1	.757	5.26	1	.031	1.83	1	.189
	Post	31.89	4.77	37.38	5.13									
	d	−.01		.11										
Eating Disorder Inventory (EDI-2)														
Drive for thinness	Pre	4.59	.81	4.39	.79	11.03	1	.002	3.05	1	.090	15.34	1	<.001
	Post	3.2	.80	4.32	.92									
	d	−1.72		−.08										
Bulimia	Pre	3.53	.57	3.22	.73	7.95	1	.008	.015	1	.905	4.09	1	.052
	Post	2.69	.66	3.06	.98									
	d	−1.36		−.18										
Body dissatisfaction	Pre	5.31	.65	5.41	.58	8.81	1	.006	8.36	1	.007	16.71	1	<.001
	Post	4.32	.67	5.40	.71									
	d	−1.5		−.01										
Eating Disorder Examination–Questionnaire (EDE-Q)														
Restraint	Pre	2.54	1.24	1.82	1.03	.25	1	.618	1.91	1	.176	.92	1	.344
	Post	2.05	1.36	1.76	1.04									
	d	−.37		−.05										
Eating concern	Pre	2.52	1.04	2.27	1.07	11.78	1	.002	1.02	1	.32	9.18	1	.005
	Post	1.20	.99	2.30	1.31									
	d	−1.3		.02										
Weight concern	Pre	3.77	1.09	3.81	1.04	7.83	1	.009	4.59	1	.04	6.56	1	.015
	Post	2.42	1.24	3.85	1.21									
	d	−1.15		.03										
Shape concern	Pre	4.45	1.00	4.43	1.14	14.3	1	.001	9.46	1	.004	22.96	1	<.001
	Post	2.49	1.01	4.48	0.97									
	d	−1.95		.04										
Body Checking Questionnaire (BCQ)														
General Score	Pre	31.53	18.5	22.21	11.8	2.15	1	.152	.4	1	.529	4.63	1	.039
	Post	23.13	10.9	22.42	16.2									
	d	−.55			.01									
Rosenberg Self-Esteem Scale (RSES)														
General Score	Pre	29.00	6.48	25.73	6.76	3.49	1	.071	6.03	1	.02	13.89	1	.001
	Post	34.46	5.93	25.57	7.26									
	d	.87		−.02										
Beck Depression Inventory (BDI)														
General Score	Pre	12.93	7.60	16.36	7.63	5.49	1	.026	5.03	1	.032	7.28	1	.011
	Post	6.40	7.15	16.52	9.64									
	d	−.88		−.01										
Photo Distortion Technique														
“Real” body dimensions	Pre	103	8	109	13	.79	1	.381	6.12	1	.019	1.5	1	.229
	Post	102	10	114	16									
	d	−.1		.34										
“Felt” body dimensions	Pre	108	12	114	22	.061	1	.807	2.19	1	.149	0.227	1	.637
	Post	103	11	112	21									
	d	−.43		−.09										
“Ideal” body dimensions	Pre	78	10	79	9	1.63	1	.211	0.52	1	.476	3.64	1	.066
	Post	86	7	82	6									
	d	.92		.39										

Note: Mean score (M), standard deviation (SD), F-score (F), degree of freedom (df), probability level (p), effect size (d)

In the *Eating Disorder Inventory*–2, a significant Time × Group-interaction was found for the scales Drive for thinness and Body dissatisfaction. Follow-up *t*-tests, with an adjusted significance level after the bonferroni correction of *p* < .006, indicated a reduction on these scales from pre- to post-test in the IG (Drive for thinness: *t*(14) = 5.16, *p* < .001; Body dissatisfaction: *t*(13) = 4.85, *p* < .001) and stable values in the CG (Drive for thinness: *t*(18) = 0.41, *p* = .682; Body dissatisfaction: *t*(18) = 0.79, *p* = .938). Unfortunately, the interaction on the scale Bulimia failed to reach significance, whereas the difference between the scores at pre- and post-test still displayed a large effect size in the IG.

Similarly, in the *Eating Disorder Examination*–*Questionnaire*, a significant Time × Group-interaction was found for the scales Eating concern, Weight concern and Shape concern with significant reductions and large effect sizes from pre- to post-test in the IG on the subscales Eating concern (*t*(14) = 4.87, *p* < .001) and Shape concern (*t*(14) = 5.88, *p* < .001) and unchanged values in the CG (Eating concern: *t*(18) = −0.11, *p* = .913; Shape concern: *t*(18) = −0.20, *p* = .837). For the subscale Weight concern, the pre- and post-results did not differ significantly after applying the Bonferroni correction of the α-error (*t*(14) = 3.01, *p* = .008), but still displayed a large effect size. No significant interaction effect was observed for the subscale Restraint.

For the general score of the *Body Checking Questionnaire*, a significant Time × Group interaction was found, but post-hoc t-test failed to reach significance after the Bonferroni correction of the probability level (*t*(14) = 2.66, *p* = .018).

For the *Rosenberg Self-Esteem Scale*, the interaction effect was significant with an increase in the IG (*t*(14) = −4.27, *p* = .001) and unchanged values in the CG (*t*(18) = 0.18, *p* = .853).

Similarly, for the *Beck Depression Inventory*, the interaction effect reached significance with a symptom reduction in the IG (*t*(14) = 4.43, *p* < .001) and unchanged scores in the CG (*t*(18) = −0.96, *p* = .924).

Concerning the *Photo Distortion Technique*, the Time × Group interaction did not reach statistical significance for the patients’ estimation of their “real”, “felt” and “ideal” body dimensions. Although the Time × Group-interaction failed to reach significance for the estimations of the “ideal” body dimensions, the pre-post difference in the IG displayed a large effect size, hinting to a less slim-ideal body size at post-test compared to pre-test in the IG.

## Discussion

The present pilot study, which to our knowledge is the first randomized-controlled trial examining the effects of a body image therapy on the cognitive-affective, behavioral and perceptual component of body image disturbance in a sample of overweight females with BED, indicates a general improvement in body image disturbance after treatment. This improvement covers the cognitive-affective body image component including its facets body dissatisfaction, weight and shape concerns and drive for thinness. Concerning the behavioral and the perceptual component of body image disturbance, the results failed to reach significance. As from pre- to post-test, the BMI of patients remained stable, the symptom reduction in body image disturbance seems not to be due to a changed body weight. Therefore, these results pose a question concerning the therapeutic techniques and their mechanisms of change underlying these treatment effects.

Since the treatment program included three sessions that focused mainly on exposure techniques, it might be assumed that body image exposure is – at least partly – responsible for the intervention effects shown in the present study. Accordingly, previous research on under- and normal weight patients with AN and BN [51, 87] as well as with subclinical samples of body-dissatisfied females [51, 87, 88] point to positive effects of body exposure regarding a reduction of weight/shape concerns, body dissatisfaction or negative body-related emotions. Therefore, the data of the present study support the findings of a previous study [28] in the conclusion that this intervention technique seems not to be exclusively effective in under- and normal-weight females with eating- and body image problems, but also in overweight females with BED. Furthermore, other studies report that body image exposure is assumed to have an effect on the cognitive-affective and behavioral component of body image [89–91], although no change concerning the perceptual component was observed [54], which is in line with the results of the present study. This finding is not inevitably intuitive, as one could also expect adverse effects of body exposure in overweight persons, as the close inspection of one’s own body might also lead to an enhanced awareness of being overweight and thus deviating from the social norms of extreme slenderness in Western societies [92].

In the past, various possible mechanisms of body exposure have been discussed. The classic explanation includes the correction of a distorted view of one’s own body [53]. Since it is assumed that an overestimation of one’s own body is associated with increased body dissatisfaction [79], a reduction of this misperception by repeated feedback concerning one’s actual appearance should be followed by a more positive view of one’s own body. However, the general empirical evidence is contradictory concerning a correction of a distorted view of one’s own body size by means of body exposure. Whereas some studies in patients with AN and BN suggested a changeability of body size overestimation by means of single body exposure sessions or a more comprehensive body image therapy including body exposure [52, 53], others did not conclude this [54]. Additionally, it has to be considered that in contrast to AN and BN, there is no study to date clearly indicating that body size overestimation also occurs in BED [11]. Further, in the present study, data gained by the digital photo distortion technique did not reveal a reduction in overestimation of one’s own body size, which is line with previous research [13, 36]. According to the results of the digital photo distortion technique, the participants estimated their “real” and “felt” figure rather accurately, but wished for a slimmer ideal figure at pre-test, which is also in line with previous findings [13]. This questions the validity of the explanation of an altered body size estimation for possible effects of body exposure in overweight patients with BED.

As a second mechanism, recent models of body exposure did not focus on reduction of body size overestimation, but instead suggested that viewing one’s own body will lead to an increase in negative body-related emotions such as fear and disgust. For this reason, viewing one’s own body will generally be avoided or checked intensely [33]. It is assumed that body exposure sessions help to overcome body avoidance and checking behavior and lead to decreased negative body-related emotions. This occurs probably through habituation processes, which has been confirmed by previous research [28, 39]. Unfortunately, in addition to positive treatment effects, for example, in terms of a reduction of body dissatisfaction that might indirectly point to blunted body-related emotions after body image therapy, a reduction of body checking could not persist after the correction of the α-error. Further research is called for, as it is not clear whether the theoretical concepts and empirical findings concerning this method primarily developed and tested in AN and BN are also generalizable to overweight patients with BED.

Beyond body exposure, cognitive techniques might have contributed to the positive effects of the intervention. The cognitive techniques were conducted at the beginning of the group treatment and included an identification and modification of dysfunctional weight- and shape-related attitudes, also covering a critical reflection of the sociocultural slenderness ideal of Western cultures [33, 84, 93].

In addition to changes in body image disturbance, we observed changes in related symptom areas such as a reduction in eating concerns and depression as well as an increase in self-esteem. Although these symptom areas were not directly targeted in the present intervention, the body image therapy might have generalized to these features, as previous research indicates general associations between body image disturbance and eating pathology [42, 94], depressiveness [47] and low self-esteem [46].

In contrast, restrained eating was not changed by the intervention. However, the question of whether restrained eating is functional or dysfunctional in overweight patients with BED is not clear. On the one hand, due to being overweight, a decrease in caloric intake might be desirable for patients in order to lose weight. On the other hand, restrained eating increases the probability of binge eating behavior. Consequently, it was pointed out that restrained eating might serve as a trigger for binge eating behavior [95], at least in individuals who carry a genetic risk [96]. In contrast, other studies found that restrained eating is not associated with the frequency of binge eating episodes [97–100] or is even associated with the abstinence of binge eating after treatment [97]. In this context, the question can be raised whether our intervention might be counterproductive regarding overweight, since due to the decrease in weight and shape concerns and body dissatisfaction, patients might be less motivated to display weight-reducing behavior. However, previous research has shown that even by interventions that explicitly aim at a reduction of binge eating behavior, the induced weight loss is only marginal [57]. In addition, during the past 20 years, the frequency of binge eating in the general population has risen, while corresponding psychological distress and impairment have decreased [7]. Therefore, it seems reasonable not to focus exclusively on potentially high body weight and binge eating behavior itself, but also to help patients to feel better about their own body [59].

A major limitation of the present pilot study is the relatively small sample size, restraining the generalizability of the findings. Due to the small sample size the internal consistencies of some of the scales of the Eating Disorder Examination-Questionnaire and the scale Body dissatisfaction of the Eating Disorder Inventory turned out to be rather low, which is to be considered when interpreting the results. Since these measures are considered to be standard instruments in eating disorder research, they were included in the analysis. Furthermore, no follow-up analyses were conducted, so the duration of the positive treatment effects over time remains unknown. Since the participants of the CG were on a waiting list for a long-term individual therapy and many of them had already started their own therapy by the time the participants of the IG finished the group program, it was not possible to administer a crossover design to further examine the study results. To ensure the homogeneity of the group, only female participants were included in the group program. This leads to another limitation concerning possible gender-specific effects. Compared to other eating disorders, the gender ratio for BED is closer to being equal [101]: however, there is still a significant gender difference with about twice as many women as men suffering from BED [102, 103]. Furthermore, since Dingemans and Van Furth [104] reported that obese participants with BED suffer from a greater extent of weight concerns and more frequent binge eating than non-obese patients with BED, the significant difference in the BMI between the IG and the CG caused by the randomization procedure presents another limitation of this study. Replicating this study with groups of over-weight and normal-weight patients suffering from BED might help to better understand the extent to which BMI plays a role in the magnitude of body image disturbance in BED. This would also take into account the fact that at least 30% of people diagnosed with BED do not suffer from being over-weight or comorbid obesity, resulting in uncertain generalizability to normal-weight subjects with BED. To control for confounding effects and to ensure the homogeneity of the group, exclusion criteria included the intake of psychotropic medication during the program and diagnosis of personality disorders, which also limits the generalizability of the findings. Future research should take into account that many patients seeking treatment in an outpatient treatment center are medicated and suffer from comorbid personality disorders. Also, unfortunately we did not systematically control for compliance during all real-life exposure sessions and the exposure exercises that were conducted by the participants outside of the group-setting as part of assigned home-work. Instead, we relied on their reports and the positive effects of the treatment. Future studies should imply standard procedures to control for compliance in all parts of the intervention.

## Conclusion

The results of the present pilot study are encouraging, as they indicate that a cognitive-behavioral body image therapy is effective for overweight females with BED with relatively high effect sizes concerning all components of body image disturbance, especially when considering that the intervention was rather short in duration with ten sessions and that it was delivered in a cost-effective group format. Future research with larger sample sizes and follow-up analyses is needed to validate and hopefully extend the results of this pilot-study. Equality of BMI between the groups would further help to integrate the results into existing knowledge concerning the diagnosis and treatment of BED. Another interesting area of future research might be to examine the additive effect of this treatment component to a standard treatment.

## Abbreviations

AN: Anorexia Nervosa
BED: Binge Eating Disorder
BN: Bulimia Nervosa
CG: Control Group
DSM: Diagnostic and Statistical Manual of Mental Disorders
EDE: Eating Disorder Examination–Interview
IG: Intervention Group
SCID: Structured Clinical Interview for DSM-IV
